# Supplementary material for: Global meta-analysis shows pervasive phosphorus limitation of aboveground plant production in natural terrestrial ecosystems
Source: Nat Commun. 2020 Jan 31;11:637. doi: 10.1038/s41467-020-14492-w (PMC6994524; doi:10.1038/s41467-020-14492-w)
Supplement: Supplementary file 1 — Supplementary Information [file 41467_2020_14492_MOESM1_ESM.pdf]

Supplementary Information for

Global meta-analysis shows pervasive phosphorus limitation of aboveground plant production in natural terrestrial ecosystems

Hou et al.

Supplementary Table 1. Summary of site characteristics and fertilization regimes in the natural terrestrial ecosystems and the croplands where the effect of P addition on aboveground plant production was assessed.

Parameter	Unit	Natural terrestrial ecosystems			Croplands		
		Mean(*SD*)	Range	*N*	Mean(*SD*)	Range	*N*
Publication time	yr	2002(14)	1959–2017	436	2001(10)	1955–2017	216
Latitude	º	39.5(15.2)	-54.8–76.5	436	28.1(23.4)	-37.5–56.5	216
Longitude	º	8.6(101.6)	-159.6–175.8	436	-35.4(81.1)	-121.7–151.4	216
Altitude	m	484(751)	0–3510	436	436(461)	1–3013	216
Site slope	º	11.6(7.4)	1.4–25	28	1.72	1.72–1.72	1
Mean annual temperature	ºC	11.5(8.1)	–12.1–27.5	436	13.8(6.9)	1.7–30	216
Mean annual precipitation	mm yr^{-1}	969(653)	80–5302	436	896(371)	80–1938	216
Aridity index		1.02(0.51)	0.08–3.69	436	0.75(0.3)	0.05–2.38	216
Soil organic C	g kg^{-1}	58.5(89.4)	2.3–456.9	112	17.7(13.1)	1.7–100.2	141
Soil total N	g kg^{-1}	3.3(4.2)	<0.1–25.0	170	1(1)	0.2–7.4	54
Soil total P	mg kg^{-1}	500(593)	41.8–4776.4	59	716.2(513)	73–2125	12
Soil available P	mg kg^{-1}	20.5(27.5)	0.2–227.6	197	19.4(22.8)	0.9–171	174
Soil pH in water		5.7(1.1)	3.3–9.7	192	6.5(0.8)	4.4–8.4	169
Soil sand content	%	41.9(23.7)	1.5–93.3	38	56.8(25.9)	2–95.1	39
Soil silt content	%	34.1(18.9)	5-79	37	21.7(16.3)	3.7–70	38
Soil clay content	%	27.5(19.5)	1.0–88.7	47	23.6(14.4)	2.75–57	62
P addition rate	kg ha^{-1}	281(499)	0.2–7905	436	149(321)	7–2340	216
Experimental duration	yr	4.5(5.5)	0.2–50	436	3.2(6.9)	0.1–60	213

NA indicates not available. *SD* indicates standard deviation.

Supplementary Table 2. Summary of site characteristics and fertilization regimes in four types of natural terrestrial ecosystems.

Parameter	Unit	Forest		Grassland		Tundra		Wetland	
		Mean(*SD*)	*N*	Mean(*SD*)	*N*	Mean(*SD*)	*N*	Mean(*SD*)	*N*
Publication time	yr	1995(15)	134	2007(12)	201	1997(11)	16	2004(8)	85
Abs(Latitude)	º	30.6(13.5)	134	42.7(13.6)	201	57(14.3)	16	42.8(14.5)	85
Longitude	º	38.9(117.6)	134	18.2(93.9)	201	-97.4(61.6)	16	-42(59.5)	85
Altitude	m	565(601)	134	539(794)	201	1449(1558)	16	49(173)	85
Site slope	º	8.7(3.7)	14	16.1(8.8)	12	5(0)	2	NA	0
Mean annual temperature	ºC	15.3(5.9)	134	10.3(6.8)	201	-5.1(6.9)	16	11.5(9.2)	85
Mean annual precipitation	mm yr^{-1}	1403(830)	134	696(389)	201	563(427)	16	1005(461)	85
Aridity index		1.2(0.6)	134	0.9(0.5)	201	0.8(0.5)	16	1.1(0.2)	85
Soil organic C	g kg^{-1}	45.8(67.7)	48	41.4(50.7)	49	258.40	1	148(169.4)	14
Soil total N	g kg^{-1}	3.4(5.3)	63	2.7(2.5)	97	5.9(5.7)	2	9.3(6.7)	8
Soil total P	mg kg^{-1}	376(599)	38	639(518)	12	1195(290)	2	737(558)	7
Soil available P	mg kg^{-1}	8.5(12.8)	47	24.2(30)	142	33.9(31.5)	4	13.8(15.5)	4
Soil pH in water		5.1(1)	62	6.1(1)	116	4.7(1.2)	3	5.6(1.2)	11
Soil sand content	%	42(25)	16	42(23)	22	NA	0	NA	0
Soil silt content	%	31(21)	15	36(18)	22	NA	0	NA	0
Soil clay content	%	36(25)	20	21(10)	25	NA	0	20(14)	2
P addition rate	kg ha^{-1}	234(255)	134	281(231)	201	198(160)	16	368(1029)	85
Experimental duration	yr	6.9(7.9)	134	4(4)	201	3.3(1.8)	16	2.1(1.9)	85

NA indicates not available. *SD* indicates standard deviation.

Supplementary Table 3. There were significant P limitation cases in all groups of ecosystems which were divided by site location, climate, fertilization regimes, or ecosystem properties. Total sample size and the proportion of significant P limitation cases in total sample size are given for each group of ecosystems. Only groups with a total sample size ≥ 8 are shown.

Group	Proportion (Total sample size)		Group	Proportion(Total sample size)	
	Natural terrestrial ecosystems	Croplands		Natural terrestrial ecosystems	Croplands
**All**	45%(436)	48.6%(216)	**Ecosystem type**		
**Continent**			Forest	52.2%(134)	NA
Australia	61.4%(101)	58.3%(12)	Grassland	36.8%(201)	NA
Asia	52.9%(34)	40%(25)	Tundra	43.8%(16)	NA
South America	53.3%(15)	12.5%(8)	Wetland	52.9%(85)	NA
North America	45.8%(153)	17.2%(128)	**Forest age (yr)**		
Europe	30%(110)	25%(8)	≤5	43.8%(32)	NA
Africa	21.7%(23)	54.3%(35)	5-20	58.7%(46)	NA
**Climate zone**			20-65	57.6%(33)	NA
Tropic	51.6%(62)	75%(40)	Mature	45.5%(22)	NA
Subtropic	55.1%(98)	61.1%(36)	**Forest composition**		
Temperate	50.3%(157)	36.7%(128)	Pure	52.4%(105)	NA
(Sub)arctic	26.1%(119)	50%(12)	Mixed	51.7%(29)	NA
**Altitude**			**Crop species**		
≤1000 m	46.4%(371)	46.1%(191)	Beans		40.3%(77)
>1000 m	36.9%(65)	68%(25)	Corn		31%(42)
**Aridity**			Vegetable		8.3%(12)
Humid	44.1%(229)	37.5%(32)	Wheat		76.3%(38)
Sub-humid	50%(108)	41.4%(116)	**Soil weathered extent**		NA
Dry subhumid	53.7%(41)	72.7%(22)	Strongly	61.2%(67)	78.6%(14)
Semi-arid	32.7%(49)	68.4%(38)	Intermediately	50%(50)	41.9%(129)
Arid	33.3%(9)	37.5%(8)	Slightly	53.6%(56)	56%(25)
**P addition amount (kg ha^{-1})**			**Soil type**		
>500	54.4%(68)	84.6%(13)	Alfisols	63.2%(19)	58.3%(24)
200-500	49%(104)	61.9%(21)	Andisols	58.8%(17)	NA
50-200	44%(191)	45.9%(74)	Aridisols	37.5%(8)	NA
≤50	32.9%(73)	43.5%(108)	Entisols	33.3%(15)	NA
**Experimental duration (yr)**			Inceptisols	62.5%(24)	57.1%(14)
>10	75%(28)	75%(16)	Mollisols	45.5%(22)	30.8%(91)
5-10	46.2%(65)	75%(4)	Oxisols	88.9%(9)	75%(8)
1-5	42.3%(248)	66.7%(42)	Spodosols	55.6%(27)	NA
≤1	42.1%(95)	39.1%(151)	Ultisols	58.1%(31)	NA
**Fertilizer type**			Vertisols		85.7%(14)
Single superphosphate	60.9%(138)	84.6%(39)	**Parent material**		
Triple superphosphate	44.6%(148)	52.3%(65)	Acid	44.1%(34)	NA
Others	30.7%(150)	33.9%(112)	Calcareous	73.7%(19)	NA
**Plant function**			Intermediate	67.7%(31)	NA
Non-N-fixing	43.9%(296)	50%(124)	Mafic	40%(15)	NA
Mixed	62.5%(24)	33.3%(3)			
N-fixing	58.6%(29)	47.2%(89)			

NA indicates not available.

Supplementary Table 4. Summary of gap filling for calculating the inverse variance.

Treatment	Sample size of the experiment		*SD* of aboveground plant production	
	Missing *N* (proportion)	Filled value	Missing *N* (proportion)	Filled value (% of the mean)
**Natural terrestrial ecosystems**				
Control	50(11.3%)	4	233(53.4%)	35.5
P treatment	50(11.3%)	4	234(53.7%)	31.1
**Croplands**				
Control	11(5.1%)	4	196(90.7%)	15.4
P treatment	11(5.1%)	4	196(90.7%)	14.3

SD indicates standard deviation.

Supplementary Table 5. P effect size grouped by the measure of aboveground plant production.

Measure of aboveground plant production	*N*	P effect size (%)	Lower *CI* (%)	Upper *CI* (%)
**Forest ecosystems**				
Aboveground plant biomass production	33	78.5	45.1	119.6
Diameter increase	34	31.8	17.9	47.4
Height increase	16	24.3	-0.9	55.8
Basal area increase	25	38.6	22.9	56.3
Volume increase	25	34.3	13.3	59.3
Litterfall production	1	26.0	-26.4	115.8
**Grasslands**				
Aboveground plant biomass production	201	29.4	26.8	32.1
**Tundra**				
Aboveground plant biomass production	10	13.6	-5.7	36.9
Leaf mass per tiller	4	89.8	51.1	138.3
Tiller biomass	1	4.3	-36.1	70.2
Plot level NDVI	1	25.7	-8.8	73.3
**Wetlands**				
Aboveground plant biomass production	72	39.4	29.1	50.5
Height increase	5	147.1	57.0	289.0
Leaf area index	3	81.5	-25.4	441.3
Production of whole plants	3	38.9	-28.5	169.9
Chamber based GPP	2	10.7	-20.2	153.5
**Croplands**				
Aboveground plant biomass production	85	16.5	10.9	22.3
Marketable yield	131	13.1	9.8	16.4

Note: *CI* indicates confidence interval.

Supplementary Table 6. A summary of the quality check of this meta-analysis. Quality criteria are derived from Koricheva and Gurevitch[1].

Quality criteria	This study
1. Has formal meta-analysis been conducted (i.e. combination of effect sizes using standard meta-analytical methodology) or is it simply a vote count?	Yes, both formal meta-analysis and vote count were used. Formal meta-analysis with five meta-analytic methods were used at the global scale and only the method of Ln(Response Ratio) weighted by inverse variance was used for all the other meta-analyses (e.g. in a region). Vote count was used to map the global distribution of significant P limitation cases
2. Are details of bibliographic search (electronic data bases used, keyword combinations, years) reported in sufficient detail to allow replication?	Yes, all electronic data (including extracted data and reference list) used in the study are provided. And a PRISMA flow diagram is also provided.
3. Are criteria for study inclusion/exclusion explicitly listed?	Yes, all criteria are listed in the Methods section.
4. Have standard metrics of effect size been used or, if nonstandard metrics have been employed, is the distribution of these parameters known and have the authors explained how they calculated variances for such metrics?	Yes, we used standard metrics of effect size, i.e., Ln(Response Ratio) weighted by inverse variance, and effect size (%) is also calculated.
5. If more than one estimate of effect size per study was included in the analysis, has potential non-independence of these estimates been taken into account?	Yes, only one estimate was retained in each experiment. An experiment is a temporally and spatially distinct experiment with internally consistent controls. One publication can have more than one experiment.
6. Have effect sizes been weighted by study precision or has the rational for using unweighted approach been provided?	Yes, effect sizes were weighted by the inverse variance. And effect sizes at the global scale were also weighted uniformly, by P addition amount, or by experimental duration.
7. Have statistical model for meta-analysis and the software used been described?	Yes, random-effect model in “meta” package in R version 3.3.1.
8. Has heterogeneity of effect sizes between studies been quantified?	Yes, confidence interval was given in all meta-analyses, and the accumulated percentage of effect sizes were shown in Fig. 2.
9. Have the causes of existent heterogeneity in effect sizes been explored by meta-regression?	Yes, temporal change in effect size was explored by meta-regression. The causes of heterogeneity were also explored by grouping experiments and by using a boosted regression tree method which can deal with multiple interactive predictors and both continuous and category variables. The nonlinear relationship between effect size and soil available P was explored with a traditional regression analysis.
10. If effects of multiple moderators have been tested, have potential non-independence of and interactions between moderators been taken into account?	Yes, both potential non-independence of and interactions between moderators were taken into account by the boosted regression method. Also, we did variable selections before the analysis to avoid highly non-independence of moderators.
11. If meta-analysis combined studies conducted on different species, has phylogenetic relatedness of species been taken into account?	Not applicable to this study.
12. Have tests for publication bias been conducted?	Yes, funnel plots were plotted, asymmetry test of the point distribution was performed, and trim-and-fill analysis was run for croplands, which were all shown in Supplementary Fig. 5.
13. If meta-analysis combines studies published over considerable time span, have possible temporal changes in effect size been tested?	Yes, possible temporal changes in effect size were tested using a meta-regression method, as shown in Supplementary Fig. 6.
14. Have sensitivity analysis been performed to test the robustness of results?	Yes, accumulative meta-analysis and leave-one-out meta-analysis were ran to test the robustness of results, as show in Supplementary Fig. 4.
15. Have full bibliographic details of primary studies included in a meta-analysis been provided?	Yes, as required by the journal, the full bibliographic details of primary studies are deposited in a public repository.
16. Has the data set used for meta-analysis, including effect sizes and variances/sample sizes from individual primary studies and moderator variables, been provided as electronic appendix?	Yes, as required by the journal, all the data are deposited in a public repository.

Supplementary Figure 1. Location of the field P addition experiments in this and two previous meta-analyses. (a) This study. (b) Elser *et al.*[2]. (c) Augusto *et al.*[3]. Data was not available in another two previous meta-analyses: Yue *et al.*[4] and Li *et al.*[5]. Data in brackets is the sample size in the type of ecosystem. Source data are provided as a Source Data file.

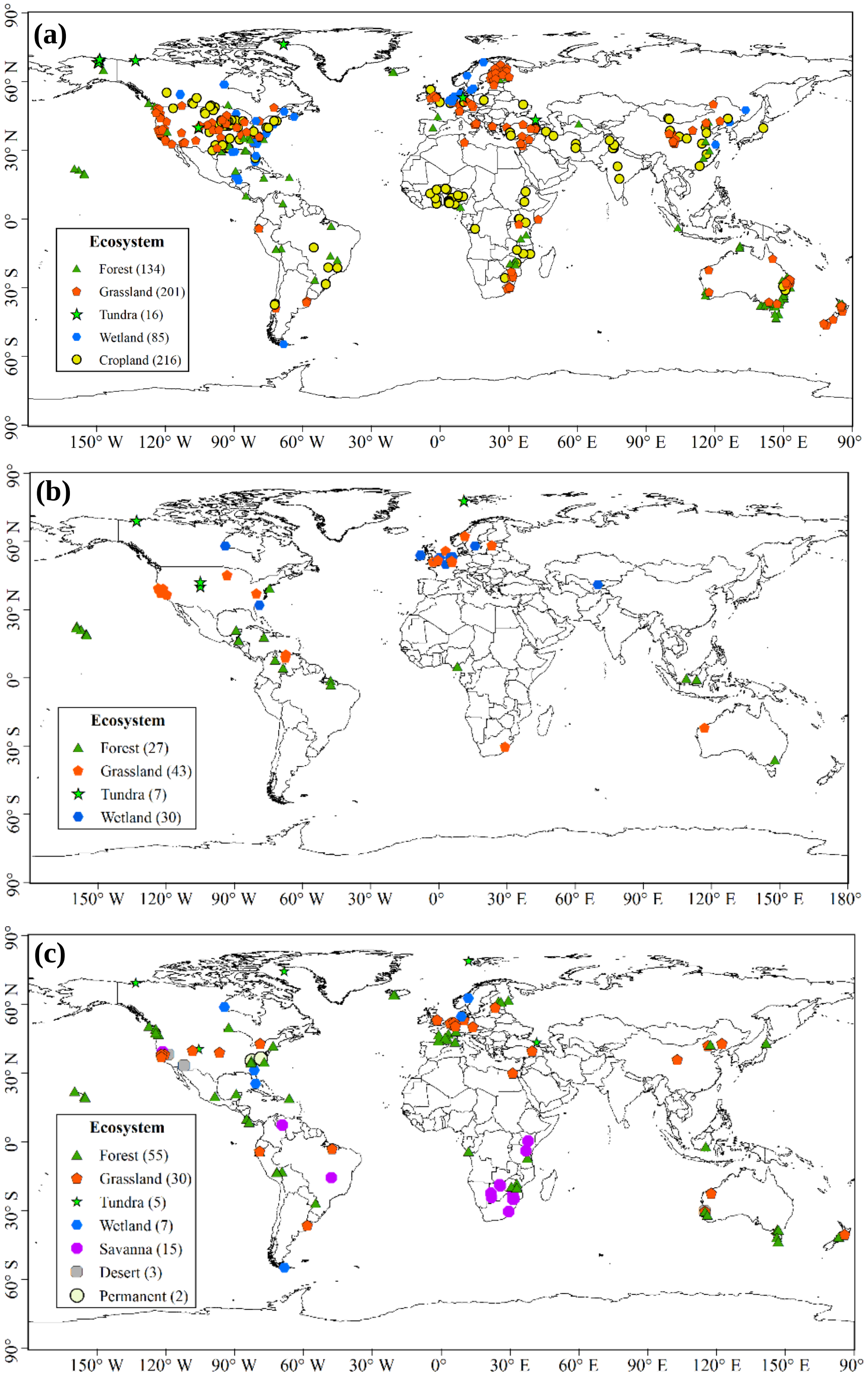

Supplementary Figure 2. Accumulated number of P addition experiments in terrestrial ecosystems with year in this and two previous meta-analyses. Source data are provided as a Source Data file.

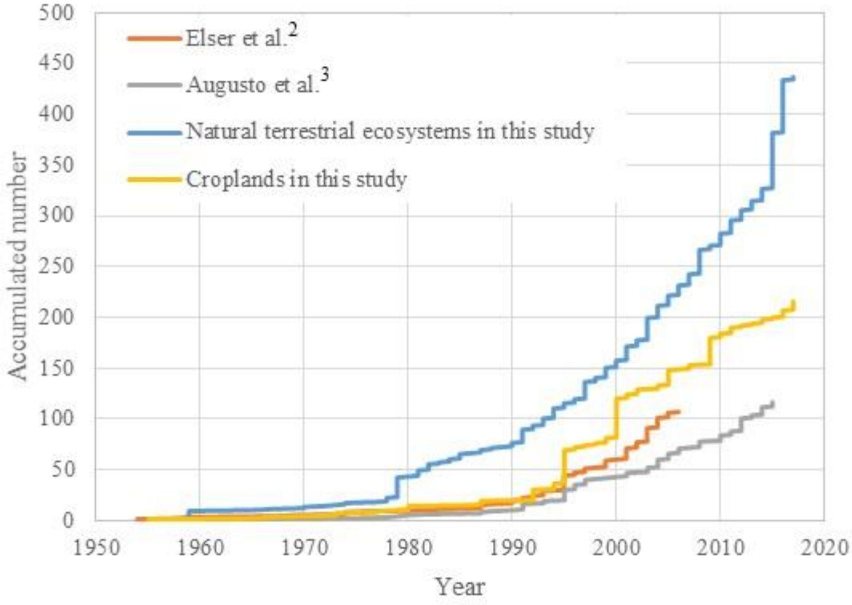

Supplementary Figure 3. Distribution of the ln transformed response ratio of aboveground plant production to P additions in the natural terrestrial ecosystems (a-c) and in the croplands (d-f), respectively. Dashed line in all subplots indicate the threshold value of Ln(Response Ratio) (0.23 in a-c, and 0.09 in d-f). Source data are provided as a Source Data file.

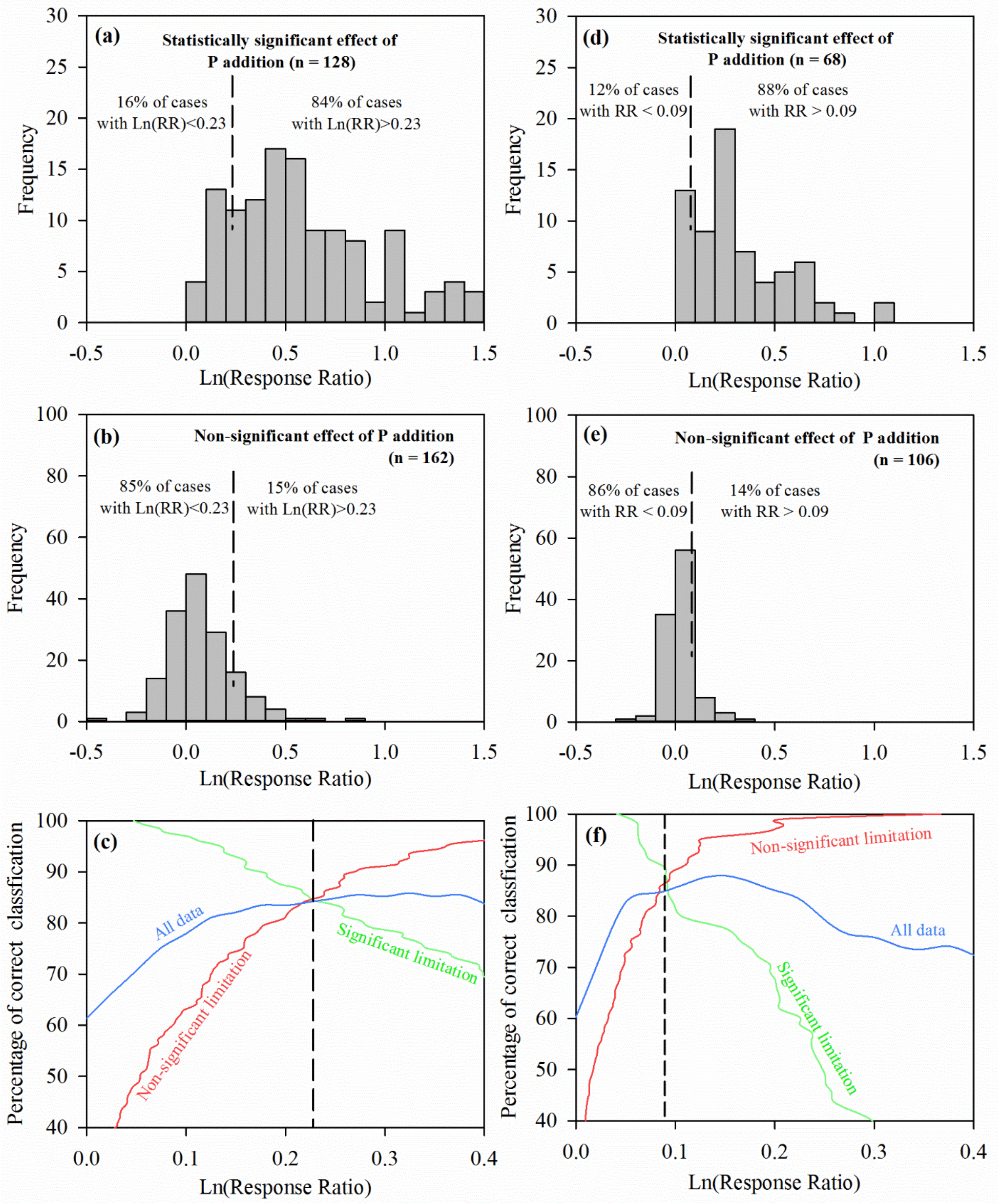

Supplementary Figure 4. Sensitivity tests of the P effect size at the global scale. Estimates of 1000 cumulative meta-analyses with random orders of experiments in the natural terrestrial ecosystems (a) and the croplands (b), respectively. (c) Leave-one-out meta-analysis in the natural terrestrial ecosystems (c) and the croplands (d), respectively. In (a) to (d), meta-analyses were performed using the method of Ln(Response Ratio) weighted by the inverse variance. (e) Estimates of 1000 cumulative meta-analyses with random orders of experiments and uniformly weighted Ln(Response Ratio) in the natural terrestrial ecosystems. The estimates in (e) were calculated to be comparable with two previous meta-analyses, i.e., Elser *et al.*[2] and Augusto *et al.*[3]. In (a) and (e), error bars indicate the confidence intervals of the estimates, which were shown as points. In (c) and (d), the black circles indicate the estimated effect sizes, the red line indicates the upper confidence interval, and the green line indicates the lower confidence interval. Source data are provided as a Source Data file.

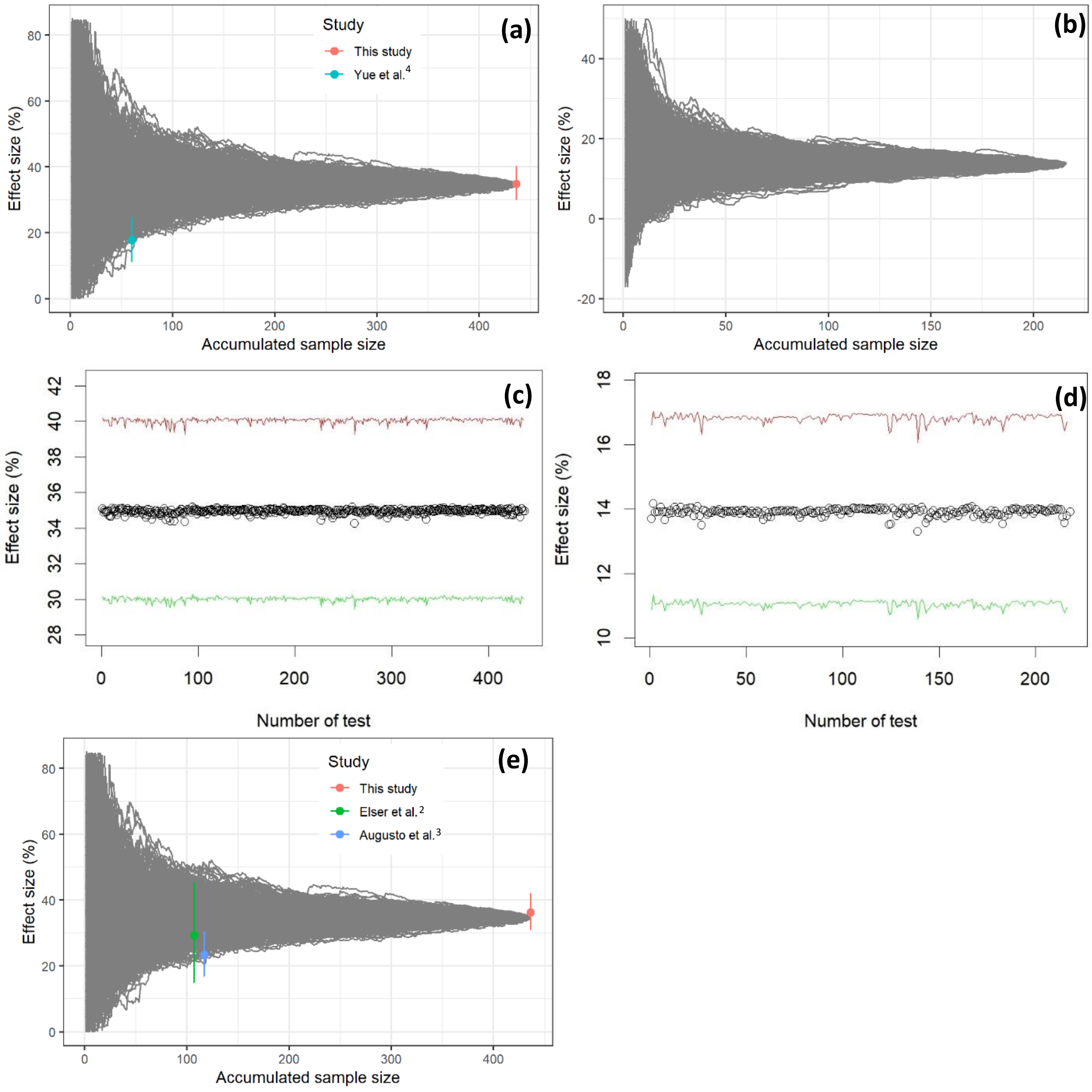

Supplementary Figure 5. Funnel plots for the effect of P addition on aboveground plant production. (a) Funnel plot in the natural terrestrial ecosystems. Asymmetry test showed a symmetric distribution (t = 1.79, P = 0.07). (b) Funnel plot in the croplands. Asymmetry test showed a positive asymmetric distribution (t = 4.23, P < 0.01). The weighted Ln(Response Ratio) decreased from 0.14[0.10, 0.16] to 0.04[0.01, 0.07] after adjusted with the trim-and-fill method. In both subplots, the outer dashed lines indicate the triangular region within which 95% of studies are expected to lie in the absence of both biases and heterogeneity. The dashed vertical line indicates the summary effect estimate. The solid vertical line corresponds to no intervention effect.

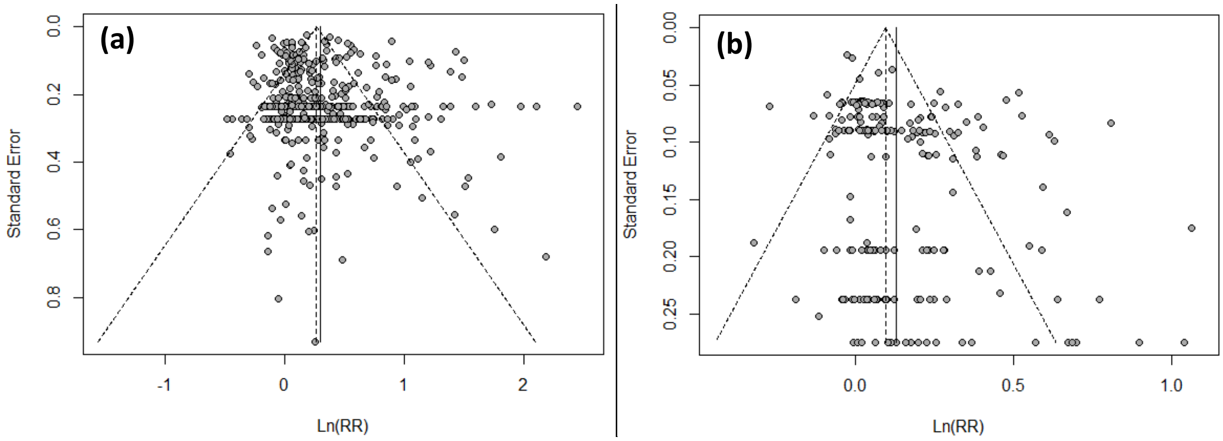

Supplementary Figure 6. A minor temporal change in P effect size with publication year in both the natural terrestrial ecosystems and the croplands. (a) Natural terrestrial ecosystems. Meta-regression analysis: Ln(Response Ratio) = 6.533 – 0.003× Publication year, $R^2 < 0.01$, $P = 0.03$, $N = 436$. (b) Croplands. Meta-regression analysis: $P = 0.18$, $N = 216$. Dot size is in proportion to weight. Source data are provided as a Source Data file.

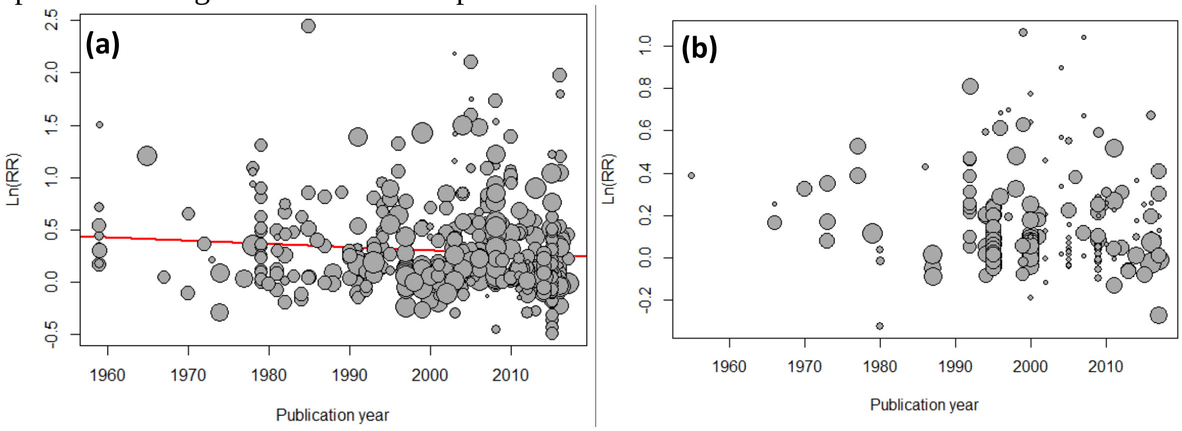

Supplementary Figure 7. Significant P limitation in all major groups of ecosystems divided by vegetation, soil, or parent material property. Exceptions are non-significant P limitation in three groups of ecosystems which had a relatively small sample size (N = 8–15). Natural terrestrial ecosystems are shown in green color and croplands are shown in yellow color, respectively. Only groups with a total sample size ≥ 8 are shown. Values represent effect sizes ± 95% confidence intervals. Point sizes are in proportion to the sample sizes, which are given in Supplementary Table 3. Dashed line indicates no P addition effect. Source data are provided as a Source Data file.

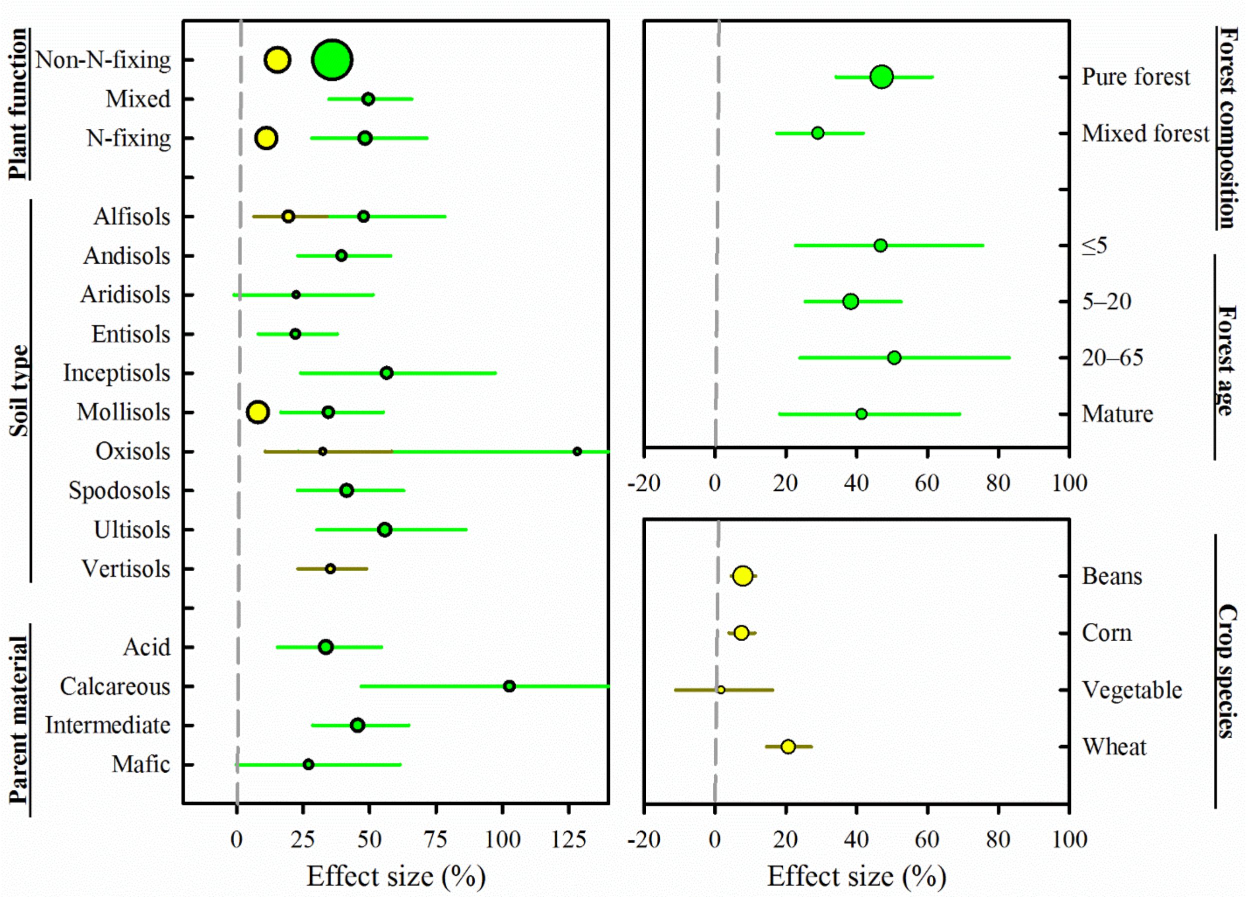

Supplementary Figure 8. The ln transformed response ratio of aboveground plant production to P additions was negatively related to the Olsen extractable P concentration in the soils. Ln(Response Ratio) = 0.48 - 0.11× Ln(Olsen extractable P), R^2 = 0.13, P < 0.001, N = 102. Data after each type of ecosystem is the averaged Olsen extractable P concentration (sample size) in the type of ecosystem. The black line and the shaded band indicate the regression line and the 95% confidence interval, respectively. If croplands are removed, the relationship becomes non-significant (linear regression, P = 0.11, R^2 = 0.07, N = 39). Source data are provided as a Source Data file.

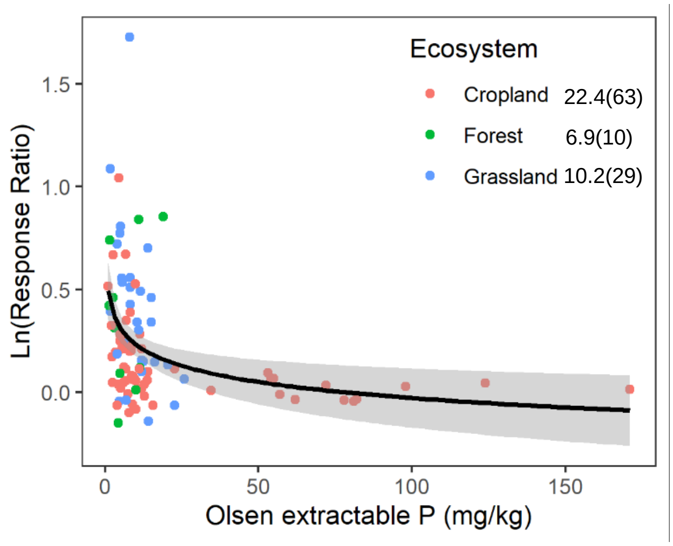

Supplementary Figure 9. Relationship between model predicted and the measured Ln(Response Ratio). (a) Relationship in the natural terrestrial ecosystems: Measured Ln(Response Ratio) = 1.30 × Predicted Ln(Response Ratio) - 0.09, $R^2 = 0.59$, $P < 0.001$, N = 436. Moran *I* test: *I* statistic = -0.65, $P = 0.74$. (b) Relationship in the croplands: Measured Ln(Response Ratio) = 1.19 × Predicted Ln(Response Ratio) - 0.03, $R^2 = 0.79$, $P < 0.001$, N = 216. Moran *I* test: *I* statistic = -1.46, $P = 0.93$. In both (a) and (b), red line is the fitted function and dashed gray line is the 1:1 line, respectively. Source data are provided as a Source Data file.

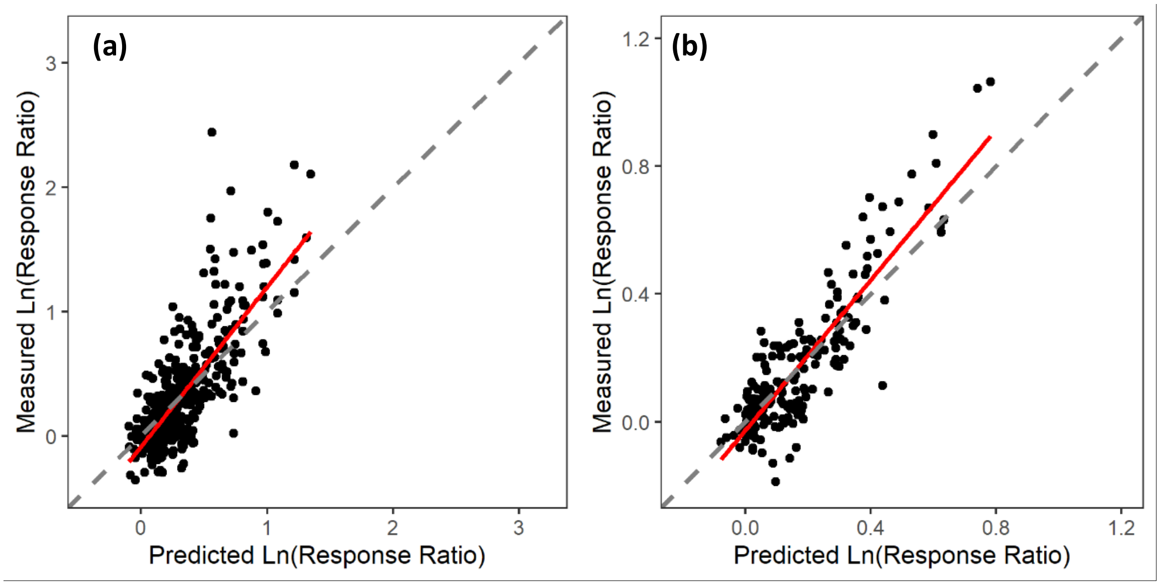

Supplementary Figure 10. A boosted regression tree partial dependence plot showing the effects of fertilization regimes, climate, and ecosystem properties on the relative (i.e., centralized) value of ln transformed response ratio (Ln(*RR*)) in the natural terrestrial ecosystems. Subplots indicate the effect of mean annual temperature (a), accumulated P addition amount (b), mean annual precipitation (c), soil available P concentration (d), soil organic C concentration (e), experimental duration (f), vegetation type (g), soil pH (h), fertilizer type (i), and plant N fixation (j), respectively. Fitted function is in black color and the smooth line is red color, respectively. Data in bracket in each subplot is the proportion of the total explained variation accounted for by the variable. Units of the numeric variables are the same as in Supplementary Table 1. Rugs in tops of the subplots indicate sample density.

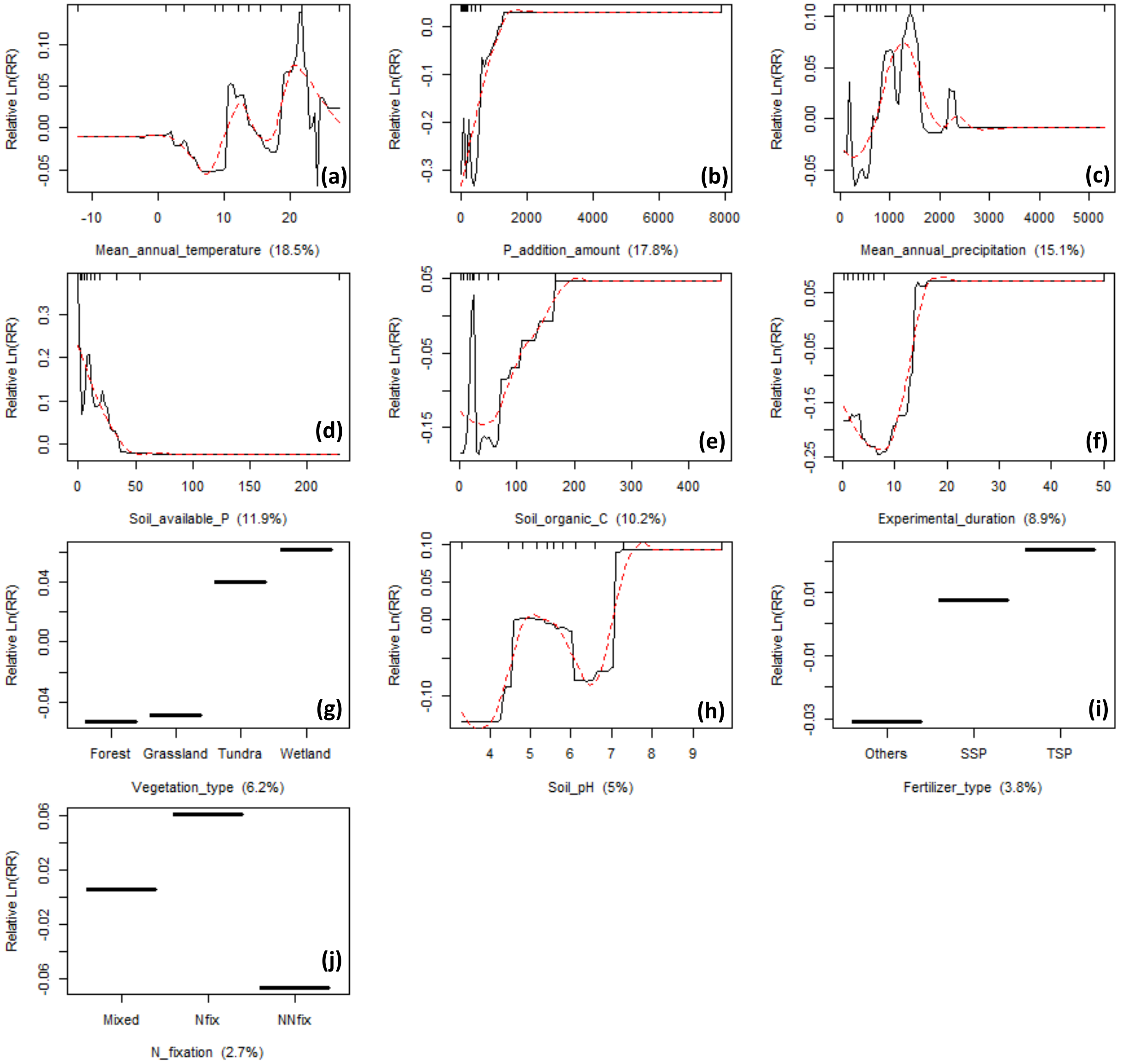

Supplementary Figure 11. A boosted regression tree partial dependence plot showing the effects of fertilization regimes, climate, and ecosystem properties on the relative (i.e., centralized) value of ln transformed response ratio (Ln(*RR*)) in the croplands. Subplots indicate the effect of soil available P concentration (a), mean annual temperature (b), accumulated P addition amount (c), crop species (d), soil organic C concentration (e), soil pH (f), mean annual precipitation (g), fertilizer type (h), experimental duration (i), and plant N fixation (j), respectively. Fitted function is in black color and the smooth line is red color, respectively. Data in bracket in each subplot is the proportion of the total explained variation accounted for by the variable. Units of the numeric variables are the same as in Supplementary Table 1. Rugs in tops of the subplots indicate sample density. In (d), (1) is Beans, (2) is Corn, (3) is Maize, (4) is Oilseeds, (5) is crops other than these in the list, (6) is Rice, (7) is Sorghum, (8) is Sugarcane, (9) is vegetable, (10) is wheat, respectively.

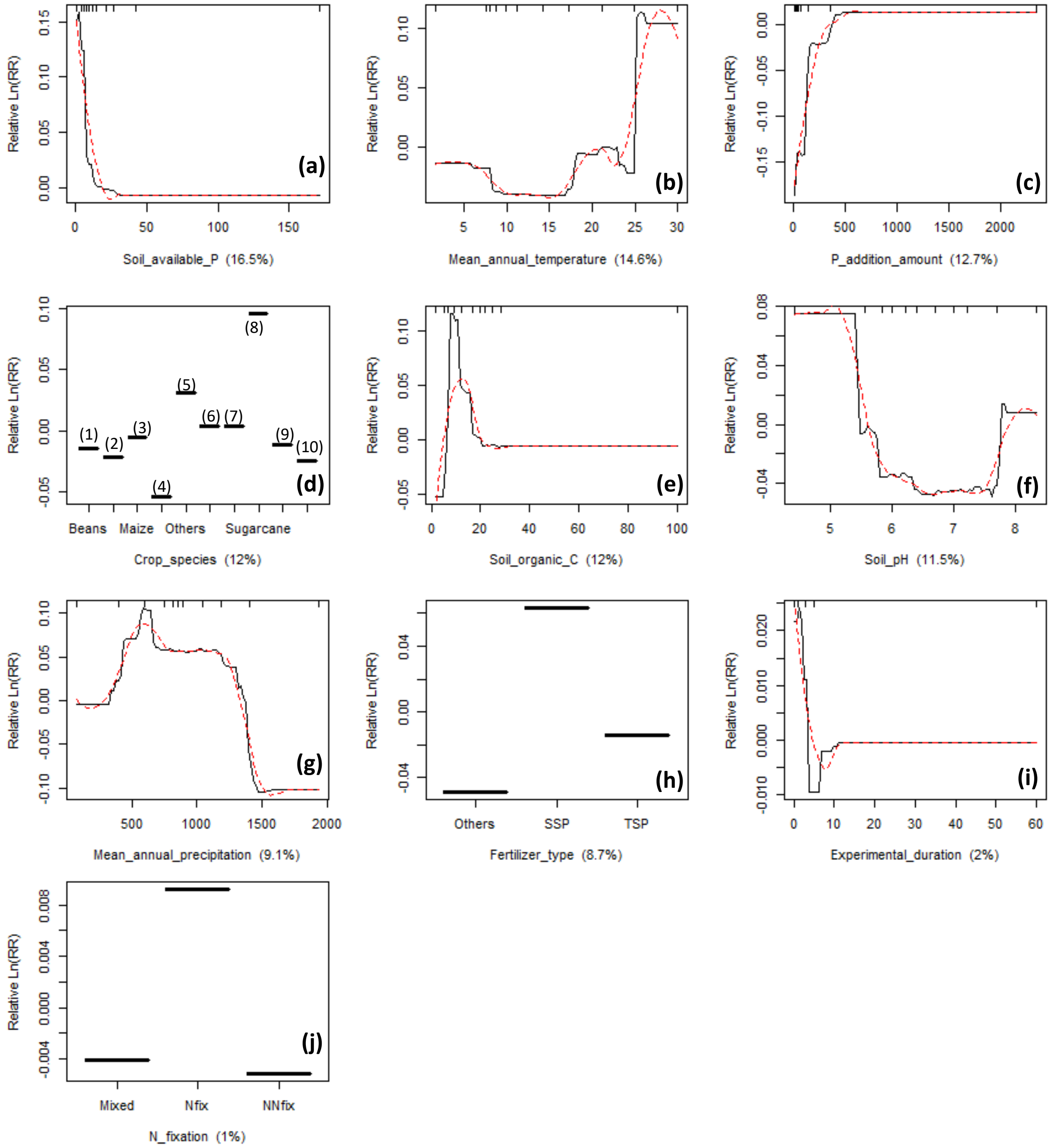

Supplementary Figure 12. PRISMA flow diagram showing the procedure used for selection of studies for synthesis.

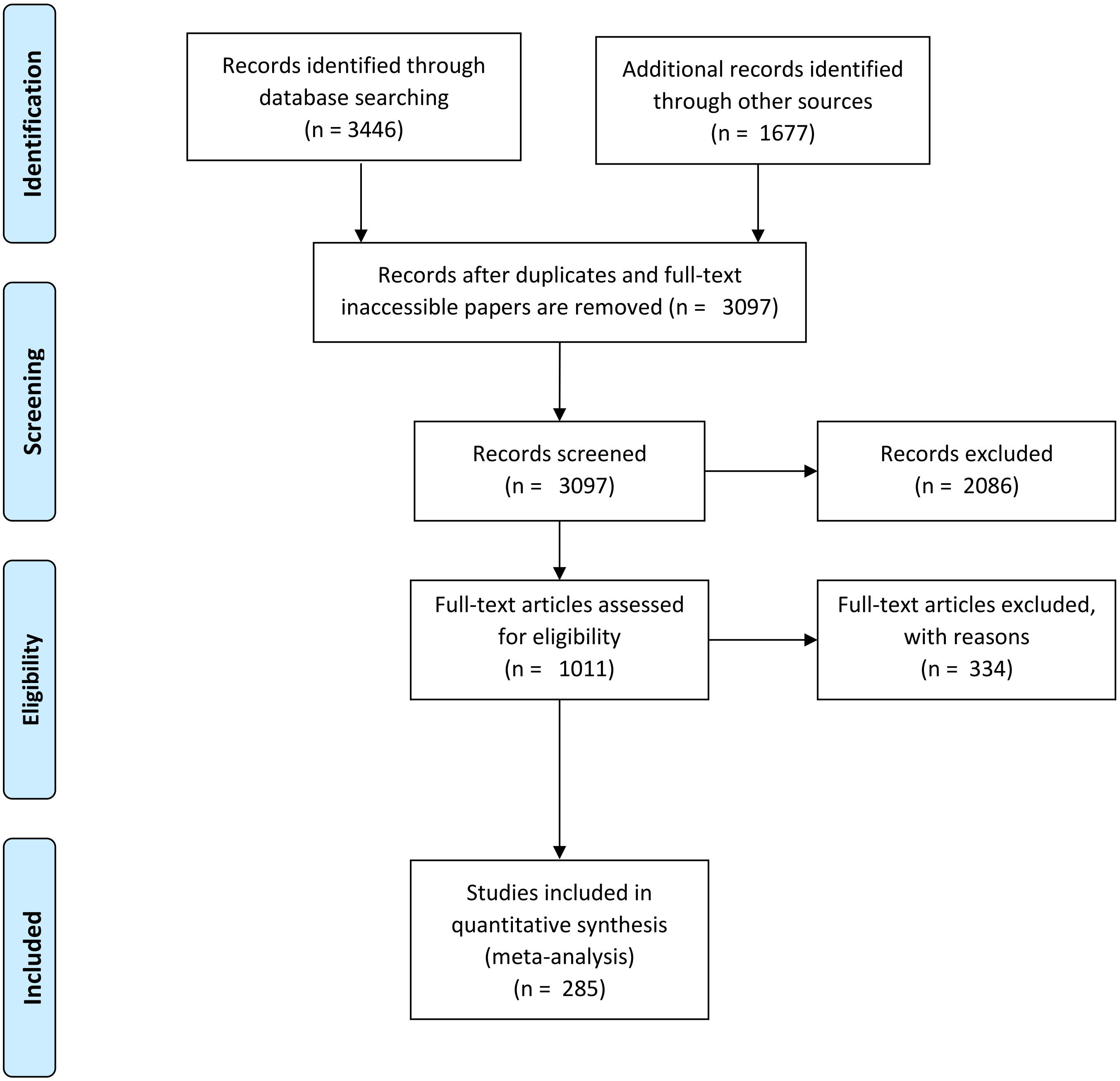

Supplementary Figure 13. The marketable yield and the aboveground plant production responded to P additions similarly in the croplands. (a) P additions did not significantly change the ratio of marketable yield to aboveground plant production across crop species. (b) The response ratio of marketable yield to P additions was closely positively related to the response ratio of aboveground plant production to P additions across species. (c) The overall response of marketable yield to P additions was not significantly different from that of aboveground plant production. Values represent effect sizes ± 95% confidence intervals. Source data are provided as a Source Data file.

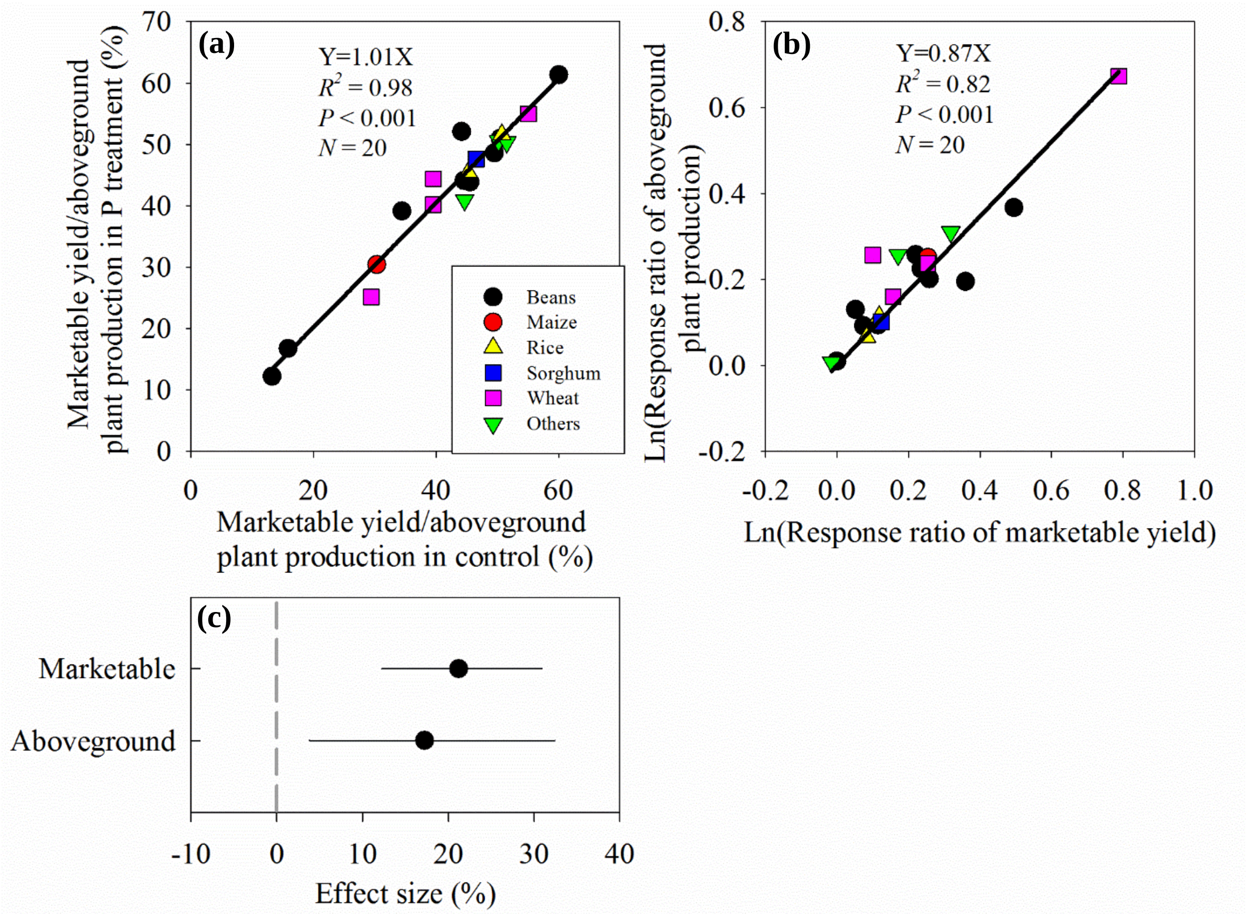

References

1. Koricheva, J. & Gurevitch, J. Uses and misuses of meta - analysis in plant ecology. *J. Ecol.* **102**, 828-844 (2014).
2. Elser, J. J. et al. Global analysis of nitrogen and phosphorus limitation of primary producers in freshwater, marine and terrestrial ecosystems. *Ecol. Lett.* **10**, 1135-1142 (2007).
3. Augusto, L., Achat, D. L., Jonard, M., Vidal, D. & Ringeval, B. Soil parent material - a major driver of plant nutrient limitations in terrestrial ecosystems. *Glob. Change Biol.* **23**, 3808-3824 (2017).
4. Yue, K. et al. Influence of multiple global change drivers on terrestrial carbon storage: additive effects are common. *Ecol. Lett.* **20**, 663-672 (2017).
5. Li, Y., Niu, S. & Yu, G. Aggravated phosphorus limitation on biomass production under increasing nitrogen loading: a meta - analysis. *Glob. Change Biol.* **22**, 934-943 (2016).
