# Supplementary material for: Global meta-analysis shows pervasive phosphorus limitation of aboveground plant production in natural terrestrial ecosystems
Source: Nat Commun. 2020 Jan 31;11:637. doi: 10.1038/s41467-020-14492-w (PMC6994524; doi:10.1038/s41467-020-14492-w)
Supplement: Supplementary file 3 — Reporting Summary [file 41467_2020_14492_MOESM3_ESM.pdf]

nature research

Corresponding author(s): Enqing Hou

Last updated by author(s): Dec 18, 2019

Reporting Summary

Nature Research wishes to improve the reproducibility of the work that we publish. This form provides structure for consistency and transparency in reporting. For further information on Nature Research policies, see Authors & Referees and the Editorial Policy Checklist .

Statistics

For all statistical analyses, confirm that the following items are present in the figure legend, table legend, main text, or Methods section.

n/a	Confirmed	
☐	☒	The exact sample size (*n*) for each experimental group/condition, given as a discrete number and unit of measurement
☐	☒	A statement on whether measurements were taken from distinct samples or whether the same sample was measured repeatedly
☐	☒	The statistical test(s) used AND whether they are one- or two-sided<br>*Only common tests should be described solely by name; describe more complex techniques in the Methods section.*
☐	☒	A description of all covariates tested
☐	☒	A description of any assumptions or corrections, such as tests of normality and adjustment for multiple comparisons
☐	☒	A full description of the statistical parameters including central tendency (e.g. means) or other basic estimates (e.g. regression coefficient) AND variation (e.g. standard deviation) or associated estimates of uncertainty (e.g. confidence intervals)
☐	☒	For null hypothesis testing, the test statistic (e.g. *F*, *t*, *r*) with confidence intervals, effect sizes, degrees of freedom and *P* value noted<br>*Give P values as exact values whenever suitable.*
☒	☐	For Bayesian analysis, information on the choice of priors and Markov chain Monte Carlo settings
☐	☒	For hierarchical and complex designs, identification of the appropriate level for tests and full reporting of outcomes
☐	☒	Estimates of effect sizes (e.g. Cohen's *d*, Pearson's *r*), indicating how they were calculated

Our web collection on statistics for biologists contains articles on many of the points above.

Software and code

Policy information about availability of computer code

Data collection	GetData Graph Digitizer version 2.22
Data analysis	“meta” package, “gbm” package version 2.1.5, and “spdep” package version 0.7.7 in R version 3.3.1, Excel 2016

For manuscripts utilizing custom algorithms or software that are central to the research but not yet described in published literature, software must be made available to editors/reviewers. We strongly encourage code deposition in a community repository (e.g. GitHub). See the Nature Research guidelines for submitting code & software for further information.

Data

Policy information about availability of data

All manuscripts must include a data availability statement. This statement should provide the following information, where applicable:

- Accession codes, unique identifiers, or web links for publicly available datasets
- A list of figures that have associated raw data
- A description of any restrictions on data availability

All code and data used in this study, including raw data and source data underlying figures, has been deposited in Figshare: https://doi.org/10.6084/m9.figshare.8969963, as stated in Data availability and Code availability section.

Field-specific reporting

Please select the one below that is the best fit for your research. If you are not sure, read the appropriate sections before making your selection.

☐ Life sciences ☐ Behavioural & social sciences ☒ Ecological, evolutionary & environmental sciences

Ecological, evolutionary & environmental sciences study design

All studies must disclose on these points even when the disclosure is negative.

Study description	This study compiled a comprehensive database of phosphorus fertilization experiments in global terrestrial ecosystems, including both natural terrestrial ecosystems and croplands. The aim is to deliver a best-ever view of the distribution, magnitude, and drivers of phosphorus limitation of terrestrial plant production. We suggest that the role of altered P supply on plant production in global terrestrial ecosystems has been previously underestimated.
Research sample	In total, we collected data from 652 P addition experiments reported in 285 published papers, which include 436 from natural terrestrial ecosystems and 216 from croplands.
Sampling strategy	With the aim of constructing a comprehensive database of the experimentally determined effects of P additions on aboveground biomass production in global terrestrial ecosystems, we tried to collect as many experiments that fulfill our criteria (described below) as possible. Relevant studies were identified by searching ISI Web of Knowledge, Google Scholar, and China Knowledge Resource Integrated Database using combinations of key words such as "phosph* addition", "phosph* fertili*", "phosph* enrich*", "aboveground biomass", "primary product*", "crop yield", and "grain yield". Our survey also included studies summarized in previously published syntheses and the subsequent relevant studies citing those syntheses.
Data collection	Enqing Hou collected all the data. The PRISMA flow diagram (Supplementary Fig. 12) showed the procedure we used for selection of studies.
Timing and spatial scale	Timing scale: data published before July, 2017, as early as data published in 1955.<br>Spatial scale: global terrestrial ecosystems, classified into forest, grassland, tundra, wetland, and cropland.
Data exclusions	Studies applied P fertilizers such as ammonium phosphate and manure were not included, because the P fertilizers contain N that may significantly confuse the effect of P.<br>When multiple measures were reported over time at a single experimental site, we used only the latest measure.<br>When multiple levels of P fertilizer treatments were reported, we used the measure with the highest amount of P addition. Choosing the latest measure and the highest P addition amount increased the likelihood that P additions fulfill plant demand and overcome the sorption of P fertilizer by soils and soil microbial competition for P fertilizer22, 24.<br>When multiple forms of P fertilizers were tested, we chose the treatment of single superphosphate or triple superphosphate if available.<br>Single-species responses were eliminated unless drawn from a mono-dominant community.<br>Experiments in forest or savanna ecosystems that only reported the response of understory or herbaceous response to P additions were eliminated.<br>Experiments with only stand biomass responses were eliminated unless they can be used to calculate aboveground biomass production.
Reproducibility	Most of the experiments in the database reported the number of replicate and the standard deviation/error.<br>Every step of statistical analysis is described.<br>All raw data and source references are deposited in Figshare: https://doi.org/10.6084/m9.figshare.8969963.
Randomization	Samples were grouped by locations, climate conditions, fertilization regimes, and ecosystem properties, as described in detail in the Methods section.
Blinding	We are blinded to group allocation mainly through three ways. Firstly, we set explicit rules of including a study before compiling the data from the published studies, as stated clearly in the Method section. Secondly, our database included all data in the accessible and published studies that fullfiled our rules of including an experiment, without the exclusion of any of the experiments for the statistical analyses. For statistical analyses, we grouped the data all by the established or commonly used ways (e.g., the grouping of soil types according to soil weathered extent).

Did the study involve field work? ☐ Yes ☒ No

Reporting for specific materials, systems and methods

We require information from authors about some types of materials, experimental systems and methods used in many studies. Here, indicate whether each material, system or method listed is relevant to your study. If you are not sure if a list item applies to your research, read the appropriate section before selecting a response.

Materials & experimental systems

n/a	Involved in the study
☒	☐ Antibodies
☒	☐ Eukaryotic cell lines
☒	☐ Palaeontology
☒	☐ Animals and other organisms
☒	☐ Human research participants
☒	☐ Clinical data

Methods

n/a	Involved in the study
☒	☐ ChIP-seq
☒	☐ Flow cytometry
☒	☐ MRI-based neuroimaging
